# Whole genome sequencing (WGS) for food-borne pathogen surveillance and control – taking the pulse

**DOI:** 10.2807/1560-7917.ES.2017.22.23.30547

**Published:** 2017-06-08

**Authors:** Jacob Moran-Gilad

**Affiliations:** 1 Ministry of Health, Public Health Services, Israel; 2 Faculty of Health Sciences, Ben-Gurion University of the Negev, Israel; 3 ESCMID Study Group for Genomic and Molecular Diagnostics, Basel, Switzerland

Next-generation sequencing (NGS) is transforming microbiology [1]. With the increased accessibility and decrease in the costs of sequencing and the optimisation of the ‘wet laboratory’ components of NGS i.e. the quality and throughput of DNA extraction, library preparation and sequencing reactions, whole genome sequencing (WGS) of bacterial isolates is rapidly revolutionising clinical and public health microbiology. WGS is a ‘disruptive technology’ that has the potential to become a one-stop-shop for routine bacterial analysis. By replacing multiple parallel steps in the microbiology diagnostic cycle, which currently involves traditional and molecular methods, it achieves accurate and speedy species identification, inference of antimicrobial susceptibility and virulence and high-resolution subtyping [2].

Typing of food-borne pathogens was one of the earliest applications of WGS [3] and proof-of-concept has been demonstrated for the superiority of WGS over traditional typing methods such as pulsed-field gel electrophoresis (PFGE), multilocus variable-number tandem repeat analysis (MLVA) and multilocus sequence typing (MLST), for a range of high priority food-borne pathogens, including *Salmonella enterica*, *Listeria monocytogenes*, *Campylobacter* species and Shiga-toxin producing *Escherichia coli* [4]. Applications of WGS include the investigation of food-related outbreaks and surveillance to delineate the local, regional and global genomic epidemiology of pathogens and to attribute the infection source. WGS thus supports risk assessment and guides interventions for prevention and control of infections.

A growing number of (public health microbiology) laboratories and governmental agencies employ WGS in their routine practice and food-borne pathogen surveillance and even more are expected to enter this field in the near future. Thus the maturation of food-borne pathogen surveillance into the WGS era is very timely.

In order for WGS to be adopted as the new gold standard for tracking of food-borne pathogens, a key element of food-borne disease control, there is a need for robust, standardised, portable and scalable methods for analysing WGS data. However, the notable diversity of bioinformatics tools and approaches used for bacterial WGS to date, as evident from a recent survey by the Global Microbial Identifier project [5], creates a tremendous challenge for harmonising surveillance and investigation of food-borne illness, especially across geographical borders and different sectors. Calling variants based on analysis of single nt polymorphisms (SNPs) as it is being done in many food-borne outbreak investigations, offers maximal resolution and discriminatory power but is very difficult to standardise. Therefore, approaches based on gene-by-gene analyses, collectively referred to as ‘extended MLST’, such as core genome (cg) or whole genome (wg)MLST may be advantageous [6], and have been advocated in other public health settings, such as Legionnaires’ disease control [7].

PulseNet was established in the United States (US) more than 20 years ago as a laboratory network for molecular epidemiology based on standardised PFGE analysis and later expanded globally. PulseNet has been successful in engaging many players in the field of food safety on a global scale and in creating a platform for data sharing and comparison of clinical, veterinary and food isolates in over 80 countries and it has a proven track-record in supporting molecular surveillance [8]. Nevertheless, some issues remained unresolved such as creation and implementation of a global nomenclature, which is important for communicating molecular epidemiology results, both scientifically as well as operationally.

In this issue of *Eurosurveillance*, an article by Nadon et al. [9] describes the next generation of PulseNet International, which is evolving into harnessing WGS. This initiative represents a wide collaboration between many leading agencies and stakeholders in this area, including the US Centers for Disease Control and Prevention (CDC), the European Centre for Disease Prevention and Control (ECDC) and the Public Health Agency Canada (PHAC), just to name a few. The authors illustrate the technical and practical aspects of adapting the network. Notably, PulseNet International has chosen an extended MLST approach, specifically, wgMLST, as its default phylogenetic analysis tool, which should underpin a standardised and efficient nomenclature-based system. Different technical and practical aspects are reviewed and discussed, mainly focusing on information technology (IT) and bioinformatics aspects (data storage, computing power, nomenclature, data sharing), methods for validation and quality control/quality assurance. Nadon et al. highlight complexities surrounding the implementation of WGS for food-borne disease surveillance, with respect to readiness at individual country and regional levels and delineate how PulseNet plans to address these.

The evolution of PulseNet International is very encouraging and will reinforce the use of NGS in the area of food safety. That said, challenges remain that need to be addressed by the public health community. There is a need for user-friendly bioinformatics solutions that will enable automated analysis of bacterial genomes by non-experts in bioinformatics to extract valuable information in a time-efficient manner. Such solutions should offer as much backwards compatibility as possible with current typing methods since the global transition to WGS is expected to be gradual. It should also offer an efficient strain/allele nomenclature that facilitates inter-laboratory work. Moreover, bioinformatics solutions should also factor in the developments in the field of DNA sequencing, particularly long-read single molecule sequencing platforms and portable sequencing devices which are increasingly being used. While WGS of food-borne pathogens has now become the new gold standard for food-borne pathogen typing, other techniques such as strain typing and characterisation using proteomics (particularly matrix-assisted laser desorption/ionisation (MALDI) time-of-flight (TOF) mass spectroscopy) or DNA arrays are rapidly evolving and should be carefully evaluated [10]. The field of metagenomics is also rapidly advancing and culture-independent microbiology, enabling genomic analysis of pathogens directly from sequenced clinical or environmental samples (as opposed to cultured isolates), is just around the corner [11]. When laying the foundations for global food pathogen surveillance networks for the coming years, we need to be mindful of such future developments.

Different from current protocols in which only typing results are shared, the transition to genome-based surveillance inevitably involves the sharing of complete sequence data. This has many implications, not only with respect to data storage, analysis and sharing infrastructures, but also aspects such as data ownership, privacy and transparency, pertaining to both genomic sequences and the related metadata. These issues should be proactively addressed in order to provide reassurance concerning data protection and create flexible solutions that will facilitate the timely sharing of public health data by as many partners as possible.

Finally, the transition to WGS-based surveillance needs to ensure sufficient quality is maintained in order to meet national and international regulatory requirements. Nadon et al. rightfully emphasise in their paper, the importance of validation, quality control and standardisation. One major aspect in making this transition and that needs to be considered is the human factor. The successful implementation of WGS-based surveillance on a global scale requires careful planning, building of capacity and training of public health and microbiology personnel to develop local readiness, especially in limited resource settings. Care should be taken to address the ‘softer’ issues, including possible cultural, political and cross-sector barriers, which together with economical, management and operational aspects could greatly influence the successful implementation of WGS.

This is a fascinating time for public health microbiology, and initiatives such as the integration of WGS as proposed by PulseNet International, are central for leveraging recent technological advancements for the benefit of public health surveillance.
